# Analyzing Microarray Data of Alzheimer's Using Cluster Analysis to Identify the Biomarker Genes

**DOI:** 10.1155/2012/649456

**Published:** 2012-02-14

**Authors:** Satya vani Guttula, Apparao Allam, R. Sridhar Gumpeny

**Affiliations:** ^1^Department of Biotechnology, Al-Ameer College of Engineering & IT, Andhra Pradesh, Visakhapatnam 531173, India; ^2^Jawaharlal Nehru Technological University, Andhra Pradesh, Kakinada 533003, India; ^3^Endocrine & Diabetes Centre, Andhra Pradesh, Visakhapatnam 530002, India

## Abstract

Alzheimer is characterized by the presence of senile plaques and neurofibrillary tangles in cortical regions of the brain. The experimental data is taken from Gene Expression Omnibus. A hierarchical Cluster analysis and TreeView were performed to group genes on the basis of the expression pattern. The dynamic change of expression over time and diverse patterns of expression support the concept of a complex local milieu. TreeView allows the organized data to be visualized. List of 24 genes were obtained which showed high expression levels. Three genes, SORL1, APP, and APOE, are suspected to cause Alzheimer's whereas the other 21 genes are related to other diseases but may also be found to be associated with Alzheimer's, and these are TMEM59, CCT4, IGF2R, SFPQ, PRDX3, RNF14, IDS, SSBP1, SYNE2, TXNL4A, STXBP3, SMARCB1, ULK2, AGTPBP1, FABP7, CALB1, H2AFY, COPA, SAP18, ATIC and SYNCRIP.

## 1. Introduction

The brain accumulation of a neurotoxic proteolytic derivative of the amyloid precursor protein (APP) is the essential event in the pathogenesis of Alzheimer's disease (AD) leading to neuronal loss [1]. Alzheimer's disease brain pathology is also characterized by the formation of intraneuronal tau-associated neurofibrillary tangles, which cause additional neurotoxic insult since mutations in the MAPT gene encoding the tau protein are responsible for frontotemporal dementia [2]. The accumulation of neurofibrillary tangles is a feature of several neurodegenerative diseases. In some cases, this accumulation is a primary disease-causing event.

Approximately 5–10% of patients develop an early age-at-onset AD (before 65 years). The disease in up to 50% of such cases is explained by mutations in one of three genes: APP [3], presenilin 1 (PS1) [4], and presenilin 2 (PS2) [5]. Pathological mutations in these genes are responsible for an autosomal dominant trait and cause A-beta accumulation in the brain [1]. However, the pathological consequence of some mutations detected in small AD families is uncertain and needs further investigation and proposed a systematic algorithm to classify mutations in known AD genes as possibly, probably, or definitely pathogenic [6].

The genetics underlying the common late-onset form of AD is complex. The apolipoprotein E gene (APOE) is a major and well-replicated risk factor for both sporadic and familial late-onset AD [7].

People who inherit mutated APP, PS1, or PS2 genes are very likely to develop Alzheimer's disease at some point in their lives. These genes are considered predictive. However, other genes also exist and that can only influence a person's susceptibility to Alzheimer's disease, but do not mean that a person is more likely than not to develop the disease. The best studied of these is apolipoprotein E (APOE). APOE has functions throughout the body in transporting cholesterol, regulating the immune system, aiding in nerve regeneration, and metabolism.

The environmental or genetic factors are also required in order for a person to develop Alzheimer's. Genetic testing for the APOE gene is not recommended for healthy people. However, it may be a useful diagnostic test in someone with dementia.

Cluster analysis of DNA microarray data is described as statistical algorithms to arrange the genes according to similar patterns of gene expression, and the output has been displayed graphically. Hierarchical clustering is a multivariate tool often used in phylogenetics and comparative genomics to relate the evolution of species [8].

Cluster and TreeView are programs that provide a computational and graphical environment for analyzing data from DNA microarray experiments or other genomic datasets. The program Cluster can organize and analyze the data in a number of different ways. TreeView allows the organized data to be visualized and browsed. This manual is intended as a reference for using the software and not as a comprehensive introduction to the methods employed. Many of the methods are drawn from standard statistical Cluster analysis.

Independent Component Analysis as a microarray data analysis tool can also help to elucidate the molecular taxonomy of AD and other multifactorial and polygenic complex diseases [9].

## 2. Material and Methods

Cluster and TreeView are programs that provide a computational and graphical environment for analyzing data from DNA microarray experiments or other genomic datasets. The program Cluster can organize and analyze the data in a number of different ways. TreeView allows the organized data to be visualized and browsed.

Gene expression omnibus is a tool in NCBI where the data was taken. Each sample has its own accession number. The accession number can either start with GSD or GSM followed by the number. The other format is not accepted by the tool. Gene expression omnibus consist of samples in tab-delimited form which either opens in note pad or Microsoft excel.

The microarray data is downloaded from http://bioinformatics.oxfordjournals.org/ from the database-wide expression profile for individual genes, and it is possible to standardize individual gene expression intensities in a specific assay by using their unique database-wide means and standard deviations. This consideration of gene's behavior in a wide variety of biological conditions gives new insight into interpreting the expressional difference between given samples. In a biological point of a view, the expressional difference of a gene with a small database-wide expressional variation should have more attention than those with large database-wide variations.

TreeView is a program that allows interactive graphical analysis of the results from Cluster. TreeView reads in matching “*.cdt” and “*.gtr,” “*.atr,” “*.kgg,” or “*.kag” files produced by Cluster. We recommend using the Java TreeView, which is based on the original TreeView.

The sample consists of both normal and abnormal neurons which upon running in Cluster gives the final output. The final output we get has *CDT, *ATR, and *GTR file extensions. *ATR and *GTR are automatically accepted in TreeView while choosing *CDT file type.

Upon loading the file in Cluster software, the file is run in the software to get the output. The output is then run in TreeView software, and the result is obtained.

## 3. Result

**Figure 1:**
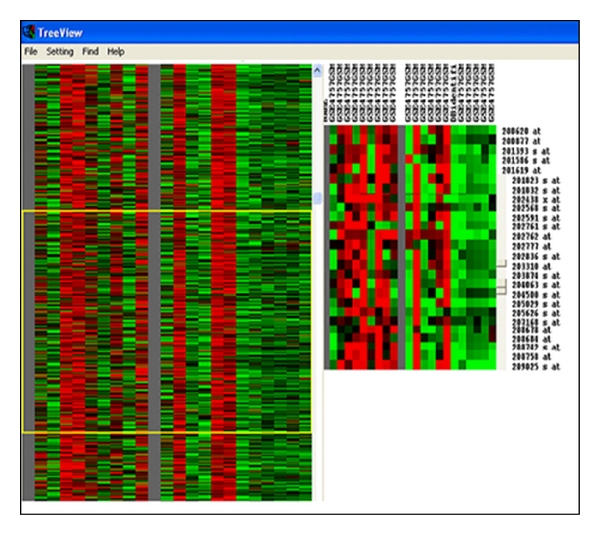
Cluster TreeView.

Cluster Analysis Reveals Distinctive Gene Expression Patterns, Figure 1, shows the analysis of entorhinal cortex neurons containing neurofibrillary tangles from 10 midstage Alzheimer's disease (AD) patients and their comparison with histopathologically normal neurons from the same patients and brain region. Results provide insight into the formation of NFTs in AD.

Twenty-six thousand and ninety-seven genes were clustered hierarchically into groups on the basis of the similarity of their expression profiles and magnitudes by the procedure of Eisen et al. [8]. The expression pattern of each gene is displayed here as a horizontal strip. For each gene, the ratio of mRNA levels in the normal neuron at the indicated time point to its level in the neurofibrillary tangled tissue is presented by a color. Red indicates that the gene is upregulated in comparison to the control, whereas green indicates the opposite. Sets of genes that clustered together were either repressed or induced at different stage.

Out of twenty-six thousand and ninety-seven genes, there were three genes which showed abrupt increase in expression. The three genes are SORL1, APP, and APOE genes.

SORL1 gene is a neuronal sorting receptor and is genetically associated with Alzheimer's disease. SORL1 has a key physiological role in the differential sorting of APP holoprotein. The recycling of APP from the cell surface via the endocytic pathways plays a key role in the generation of A-beta peptides, and when SORL1 is underexpressed, more APP protein is sorted into A-beta-generating compartment [10].

The APP gene encodes a transmembrane precursor protein involved in nuclear signalling and has multiple isoforms generated by alternative splicing. APP could be cleaved by alpha, beta, and gamma secretases. The cleavage by the beta and gamma secretases leads to the formation of A-beta peptides—the basis of the amyloid plaques in AD brains, while the alpha secretase generates soluble not amyloidogenic APP fragments. The brain accumulation of a neurotoxic proteolytic derivative of the amyloid precursor protein (APP) (A-beta 40/42 peptides) is the essential event in the pathogenesis of Alzheimer's disease (AD) leading to neuronal loss. The apolipoprotein E gene (APOE) is a major and well-replicated risk factor for both sporadic and familial late-onset AD [7].

In spite of the above three genes, there were other twenty-one genes related to other diseases but may also be found to be associated with Alzheimer's; these are transmembrane protein 59 [11], chaperonin containing TCP1 subunit 4 (delta) [12], insulin-like growth factor 2 receptor [13], splicing factor proline/glutamine-rich [14], peroxiredoxin 3 [15], ring finger protein 14 [16], iduronate 2-sulfatase [17], single-stranded DNA-binding protein 1 [18], spectrin repeat containing, nuclear envelope 2, thioredoxin-like 4A [19], syntaxin-binding protein 3 [20], SWI/SNF-related, matrix-associated, actin-dependent regulator of chromatin, subfamily a, member 1, unc-51-like kinase 2 (*C. elegans*), ATP/GTP-binding protein 1, fatty-acid-binding protein 7, brain [21], calbindin 1, 28 kDa [22], H2A histone family, member Y [23], coatomer protein complex, subunit alpha [12], Sin3A-associated protein, 18 kDa [24], 5-aminoimidazole-4-carboxamide ribonucleotide formyltransferase/IMP cyclohydrolase, and synaptotagmin-binding, cytoplasmic RNA-interacting protein [25] twenty-one genes which showed high expression level, and the genes are listed in Table 1.

## 4. Conclusion and Discussion

We have focused on presenting an overview of hierarchical clustering of microarray data, emphasizing the relationship between a dendrogram and spatial representations of genes. We believe this relationship provides an intuitive understanding of how to analyze microarray data and can make it easier to interpret the results of a Cluster analysis in a biological framework. The fact that the “heat maps” found in the majority of microarray publications are based on hierarchical clustering indicates that an understanding of this general method is valuable to those who are just beginning to read the microarray literature and even to those who are using supervised methods. We have used Cluster analysis software which is available online at Eisen laboratories and the version is Cluster 3.0.

According to Kong et al. [9], independent component analysis may contribute to a deeper understanding of gene expression data. Particularly, ICA resolves expression data at a higher resolution than is achieved by approaches based on correlations alone, even though the aim of the present investigation was not to evaluate specific ICA algorithms and procedures for analyzing microarray data. However, choosing the appropriate algorithms for analysis is a crucial element of the experimental design and will affect the type of information that is retrieved. In the present study of hierarchical clustering, we concentrated on identifying the biomarker genes associated with Alzheimer's from the microarray data.

Identification of candidate genes could provide easily accessible biomarkers to monitor Alzheimer's, and these are SORL1, and APP, and APOE, the other 21 genes listed are related to other diseases but may also be found to be associated with Alzheimer's; these are transmembrane protein 59 [11], chaperonin containing TCP1 subunit 4 (delta) [12], insulin-like growth factor 2 receptor [13], splicing factor proline/glutamine-rich [14], peroxiredoxin 3 [15], ring finger protein 14 [16], iduronate 2-sulfatase [17], single-stranded DNA-binding protein 1 [18], spectrin repeat containing, nuclear envelope 2, thioredoxin-like 4A [19], syntaxin-binding protein 3 [20], SWI/SNF-related, matrix-associated, actin-dependent regulator of chromatin, subfamily a, member 1, unc-51-like kinase 2 (*C. elegans*), ATP/GTP-binding protein 1, fatty-acid-binding protein 7, brain [21], calbindin 1, 28 kDa [22], H2A histone family, member Y [23], coatomer protein complex, subunit alpha [12], Sin3A-associated protein, 18 kDa [24], 5-aminoimidazole-4-carboxamide ribonucleotide formyltransferase/IMP cyclohydrolase, and synaptotagmin-binding, cytoplasmic RNA-interacting protein [25].

## Figures and Tables

**Table 1:** List of genes of high expression in microarray data.

AFFYMETRIX_3PRIME_IVT_ID	Gene name	Disease caused
200620_at	Transmembrane protein 59	Alzheimer's disease
200877_at	Chaperonin containing TCP1, subunit 4 (delta)	Hereditary sensory neuropathy
201393_s_at	Insulin-like growth factor 2 receptor	Parkinson's disease
201586_s_at	Splicing factor proline/glutamine-rich (polypyrimidine tract-binding protein associated)	Papillary renal cell carcinoma
201619_at	Peroxiredoxin 3	Oxidative stress to cells—Alzheimer'sand Parkinson's disease
201823_s_at	Ring finger protein 14	Insulin Resistance Syndrome
202438_x_at	Iduronate 2-sulfatase	Hunter syndrome
202591_s_at	Single-stranded DNA-binding protein 1	Crown gall disease
202761_s_at	Spectrin repeat containing, nuclear envelope 2	Cerebellar ataxia type 1
202836_s_at	Thioredoxin-like 4A	Niemann-Pick disease
203310_at	Syntaxin-binding protein 3	Cardiovascular disease
203874_s_at	SWI/SNF related, matrix-associated, actin-dependent regulator of chromatin, subfamily a, member 1	Autosomal recessive pleiotropic disorder
204063_s_at	unc-51-like kinase 2 (C. elegans)	Polycystic kidney disease
204500_s_at	ATP/GTP-binding protein 1	Musculoskeletal disease
205029_s_at	Fatty-acid-binding protein 7, brain	Coronary disease
205626_s_at	Calbindin 1, 28 kDa	Parkinson's disease
207168_s_at	H2A histone family, member Y	Ataxia-Telangiectasia
208684_at	coatomer protein complex, subunit alpha	Protein energy malnutrition
208742_s_at	Sin3A-associated protein, 18 kDa	Huntington's disease
208758_at	5-Aminoimidazole-4-carboxamide ribonucleotide formyltransferase/IMP cyclohydrolase	Non-Hodgkin's lymphoma
209025_s_at	Synaptotagmin-binding, cytoplasmic RNA-interacting protein	Alzheimer's disease
